# Risk Factors for Tungiasis in Nigeria: Identification of Targets for Effective Intervention

**DOI:** 10.1371/journal.pntd.0000087

**Published:** 2007-12-05

**Authors:** Uade Samuel Ugbomoiko, Liana Ariza, Ifeanyi Emmanuel Ofoezie, Jörg Heukelbach

**Affiliations:** 1 Department of Zoology, University of Ilorin, Ilorin, Nigeria; 2 Department of Community Health, School of Medicine, Federal University of Ceará, Fortaleza, Brazil; 3 Department of Zoology, University of Nigeria, Nsukka, Nigeria; 4 School of Public Health, Tropical Medicine and Rehabilitation Sciences, James Cook University, Townsville, Australia; George Washington School of Medicine, United States of America

## Abstract

**Background:**

The parasitic skin disease tungiasis (caused by the flea *Tunga penetrans*) affects resource-poor communities in Latin America, the Caribbean and sub-Saharan Africa. Prevalences in endemic areas are high, and severe pathology occurs commonly. However, risk factors for infestation have never been assessed in Africa.

**Methods and Findings:**

A cross-sectional study was conducted in Erekiti, a rural community in Lagos State (Nigeria), where tungiasis is endemic. Individuals were examined clinically for the presence of tungiasis, and a questionnaire was applied. Data from 643 individuals (86.6% of the target population) were analyzed; 252 (42.5%) were infested with *T. penetrans*. In the multivariate logistic regression analysis, presence of pigs on the compounds (adjusted odds ratio = 17.98; 95% confidence interval: 5.55–58.23), sand or clay floor inside houses (9.33; 5.06–17.19), and having the common resting place outside the house (7.14; 4.0–14.29) were the most important risk factors identified. The regular use of closed footwear (0.34; 0.18–0.62) and the use of insecticides indoors (0.2; 0.05–0.83) were protective against infestation. The population attributable fractions associated with tungiasis were: sand or clay floor inside the house (73.7%), resting usually outside the house (65.5%), no regular use of closed footwear (51.1%), and pigs on the compound (37.9%).

**Conclusion:**

The presence of tungiasis in Erekiti is determined to an important extent by a limited number of modifiable variables. Effective and sustainable intervention measures addressing these factors need to be implemented in this and other West African communities with high disease burden.

## Author Summary

Tungiasis is a parasitic skin disease caused by the sand flea *Tunga penetrans*. After penetration into the skin, the flea grows and reaches the size of a pea. The disease is a neglected public health problem in endemic areas in Latin America, the Caribbean and Africa, and causes considerable morbidity in the affected communities. We performed a study in a rural community in Nigeria to detect factors associated with tungiasis. People were examined for the presence of sand flea lesions, and a questionnaire was applied. Of the 643 individuals examined, 252 (42.5%) had tungiasis. The most important factors independently associated with the disease were: presence of pigs on the compound (adjusted odds ratio [OR] = 17.98), sand or clay floor inside house (OR = 9.33), and having the common resting place outside the house (OR = 7.14). The regular use of closed footwear (OR = 0.34) and the use of insecticides indoors (OR = 0.2) were protective factors. Our data show that tungiasis in this community is determined to an important extent by some variables that may be a target for interventions.

## Introduction

The parasitic skin disease tungiasis is caused by the permanent penetration of the female sand flea *Tunga penetrans* into the epidermis of its host. After penetration, the female undergoes a hypertrophy and reaches the size of a pea. Tungiasis has many features of a neglected tropical disease and thus can be considered as a paradigm: it is endemic in poor communities and rural areas, it is associated with stigma, and there is no commercial market for products targeting the disease [1]–[3]. The disease only sporadically affects travelers to endemic areas in South America and Africa, whereas people living in local communities commonly suffer from severe infestation and associated pathology [4],[5]. Associated pathology includes bacterial superinfection, pain, fissures hindering individuals from walking normally, as well as deformation and loss of toenails and digits [1], [6]–[8]. Tungiasis lesions have also been described to be port of entry for tetanus infection [7],[9],[10].

The sand flea originally occurred only on the American continent and the Caribbean Islands, but spread in the late 19^th^ century throughout sub-Saharan Africa and to Madagascar [1],[11]. Two recent studies from Nigeria and Cameroon indicate that still today tungiasis is a major public health problem in West Africa [12],[13].

In the past few years, the public health importance of tungiasis in resource-poor populations has been highlighted from different countries, including Brazil, Argentina, Haiti and Nigeria [7],[14],[15]. However, risk factors for infestation have only been addressed in a single study from Brazil [16], and sustainable intervention measures have never been assessed systematically. Control programs aiming at the reduction of severe morbidity are nonexistent.

Here we present the results of a cross-sectional study identifying major risk factors for tungiasis in a rural community in Nigeria. The results show that several modifiable factors, which can be addressed in control programs, are important determinants for infestation.

## Methods

### Study area

The study was conducted in Erekiti, a community located about 50 km west from Lagos, the capital of Lagos State, Nigeria.

The community can be regarded as typical for a small rural village in Western Nigeria; the characteristics have been described in detail elsewhere [13]. In brief, Erekiti has a population of about 1200 inhabitants. The community lacks appropriate urban services like health care centers, pipe-borne water and a public sewage system. Open wells and the nearby river serve as the source of water. The majority of the people walk barefooted, defecate in the surrounding bush and scatter domestic waste in the vicinity of their homes. Domestic animals (pigs, goats, chickens, dogs, cats) roam around freely.

### Study population and design

This cross-sectional study was carried out during the hot and dry season in March 2006, when the prevalence of tungiasis and parasite load are known to be highest [14]. Before the onset of the study, information meetings were held with community members. Thereafter, 50% of the community's households (142 households, 643 individuals) were randomly selected using a random number table. For this selection, census data of the community were used, obtained from the Lagos State National Population Commission.

The households were visited, and every participant was examined thoroughly for the presence of embedded sand fleas. A pre-tested structured questionnaire was applied in *egun*, the local language.

The information collected consisted of four categories: (1) socio-demographic factors (such as sex, age, education); (2) housing and associated factors (such as type of construction of the house, type of floor inside house, sanitary conditions, presence of electricity, waste disposal); (3) ownership and presence of domestic animals; (4) knowledge, attitudes and practices related to tungiasis (such as knowledge on transmission, regular use of footwear, common resting place, preventive measures, treatment). Children of 6 years and above provided information directly, while in the other cases information was obtained from the guardians. A household was revisited when a family member was absent.

Clinical examination was performed by inspecting carefully the legs, feet, hands and arms. To guarantee privacy, other topographical regions of the body were not examined. We considered this approach as acceptable, as in endemic communities more than 99% of tungiasis lesions occur on these areas [17]. At the clinical examination we considered the following findings diagnostic for tungiasis: an itching red-brownish spot with a diameter of one to three mm, a circular lesion presenting as a white patch with a diameter of four to ten mm with a central black dot, black crust surrounded by necrotic tissue, as well as partially or totally removed fleas leaving a characteristic sore in the skin [18]. Localization and number of lesions were recorded. As defined by Muehlen et al. [16], the presence of less than 5 lesions was considered as mild, of 6–30 as moderate and of more than 30 lesions as heavy infestation.

To reduce observation bias, clinical examinations and interviews were done by different persons, and the interviewer was blinded to the infestation status of the household members. All clinical examinations and interviews were done by a single person, respectively, to eliminate inter-observer bias.

Prevalence of tungiasis, parasite load and associated morbidity in the study population has been presented elsewhere in detail [13].

### Statistical analysis

Data were entered using Epi Info software (version 6.04d; Centers for Disease Control and Prevention, Atlanta, USA) and checked for entry errors by rechecking all data entries with the original data forms. Then, data were transferred to Stata® software package (version 9.0; Stata Corporation, College Station, USA) for analysis.

We applied Fisher's exact test to determine the significance of differences of relative frequencies. Variables were first analysed in a bivariate manner to identify those to be included in the unconditional logistic regression. Multivariate logistic regression using backward elimination was then performed, to calculate adjusted odds ratios for the independent association between tungiasis infestation and the explanatory variables. Only variables with a *p* value<0.3 in the Fisher's exact test were entered into the initial model, and then backward elimination was run. To remain in the model, a significance of *p*<0.05 was required. Confounding and interaction between variables were investigated by stratification and by constructing 2×2 tables. The variables entered in the logistic regression did not show any collinearity. All variables that remained in the final model are presented; odds ratios were adjusted for all other variables in this model.

Similar to Muehlen et al. [16], we assessed the population attributable fractions of factors associated with *T. penetrans* infestation. The population attributable fraction is the fraction of cases which would not have occurred in the community if the exposure had been avoided [16]. The population attributable fractions (PAF) were calculated for modifiable risk factors with high odds ratios, expressed as the percentage exposed among cases, multiplied by (OR-1)/OR. We based the calculation of the PAF on the adjusted odds ratios obtained from logistic regression analysis. As calculation of the PAF assumes that the exposure is causal and that the other risk factors remain unchanged, we calculated the PAF for those variables for which causality seemed to be likely and which are modifiable.

### Ethical considerations

The Ethical Committee of the Badagry Local Government Public Health Board, which is composed of medical and administrative personnel, approved the study, including the fact that oral consent was obtained. Before the study, the objectives and the study protocol were explained during meetings with the community leaders of Erekiti and a representative of the Ethical Committee. The community leaders also approved the study.

In accordance with local requirements, consent was obtained after explaining the objectives from all study participants, or in case of minors, from their caretakers. The statement was translated into the local language by our interpreter. The consent was witnessed by a person not involved in the study (usually a community representative). The participants signed, by thumbprinting, a spreadsheet containing details of their biodata. Data were kept strictly confidential.

## Results

Of the 643 individuals of the target population, 557 (86.6%) were encountered and participated in the study. This represents 45.2% of the total population of the community. Of the participants, 299 (53.7%) were male and 258 (46.3%) female. Illiteracy rate was high (n = 445; 77.2%), and 100 individuals (56.5%) of the adult working population had a mean monthly income<US$50. Most people lived in brick houses (n = 414; 74.3%); 277 (49.7%) had inside a floor of sand or clay, 363 (65.2%) did not have any toilet facilities, and 326 (58.5%) littered domestic waste within the house premises. Seventy two (12.9%) individuals walked barefooted.

**Figure 1:**
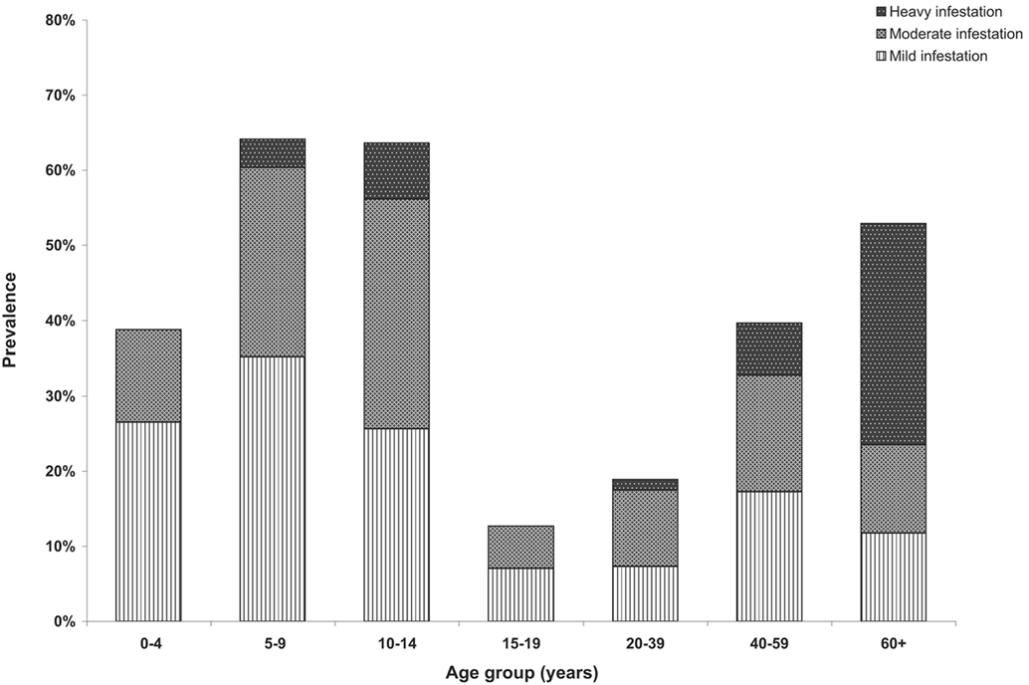
Prevalence of tungiasis stratified by age group and severity of infestation.

In total, 252 (42.5%) individuals were infested with *T. penetrans*. Of these, 122 (48.4%) presented with mild, 105 (41.7%) with moderate, and 25 (9.9%) with heavy infestation. The age-specific prevalences and intensities of infestation are shown in Figure 1. Prevalence followed an S-shaped pattern and was highest in children 5 to 9 years of age, 10 to 15-years old adolescents and the elderly. The highest proportion of individuals with heavy infestation was observed in the elderly.[Fig pntd-0000087-g002] [Fig pntd-0000087-g003]

**Figure 2 pntd-0000087-g002:**
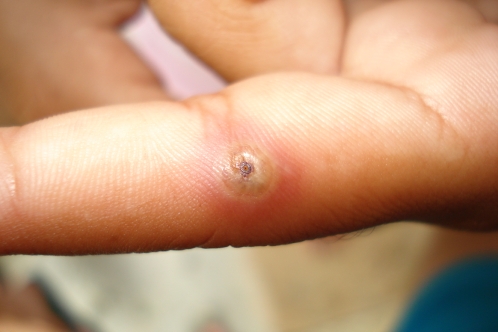
A single tungiasis lesion on the finger of a 7-year-old boy from Brazil, with surrounding erythema.

**Figure 3 pntd-0000087-g003:**
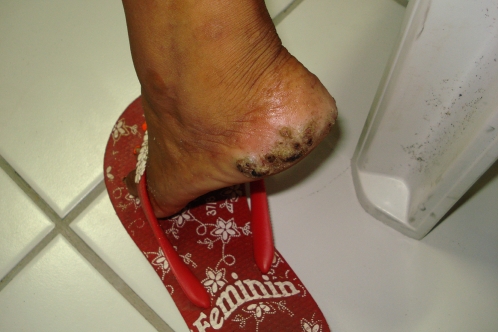
Multiple lesions on the left heel of a 13 year-old Brazilian girl.

Factors associated with tungiasis in the bivariate analysis are depicted in Table 1. Variables of all groups were detected to be associated with tungiasis. Odds ratios>10 were found for community members living in houses made of palm products; living in houses with sand or clay floor; with water supply exclusively from the river; with pigs on the compound, and those usually resting outdoors (Table 1). Other variables significantly associated with infestation included young age (OR = 9.0), low education (OR = 10.0), lack of knowledge on transmission (OR = 7.22), no regular use of footwear (OR = 5.9), no use of soap for bathing (OR = 5.9), and the non-use of commercialized insecticides used as a means of prevention (OR = 9.3).

**Table 1:** Bivariate analysis of factors associated with tungiasis infestation.

	Examined n	Positive n (%)	OR (95% confidence interval)	*P* value
**Socio-demographic factors**				
Sex				
Female	258	111 (43.0)	Reference	
Male	299	141 (47.2)	1.18 (0.83–1.68)	0.35
Age group (years)				
≤14	329	198 (60.2)	9.00 (5.34–15.58)	<0.0001
15–39	153	22 (14.4)	Reference	
≥40	75	32 (42.7)	4.43 (2.22–8.88)	<0.0001
Religion				
Christian	445	203 (45.6)	Reference	
Muslim	75	36 (48.0)	1.10 (0.65–1.85)	0.71
None/other	37	13 (35.1)	0.65 (0.29–1.36)	0.23
Education				
Primary school completed	127	14 (11.0)	Reference	
Illiterate/Primary school not completed	430	238 (55.4)	10.00 (5.48–19.43)	<0.0001
Occupation				
School	380	203 (53.4)	2.77 (1.83–4.22)	<0.0001
Worker/employed	157	46 (29.3)	Reference	
Unemployed/Retiree	20	3 (15.0)	0.42 (0.07–1.58)	0.29
**Housing and associated factors**				
Distance to river (walking time)				
<15 min	199	92 (46.2)	1.06 (0.74–1.53)	0.79
≥15 min	358	160 (44.7)	Reference	
Type of house construction				
Bricks	414	150 (36.2)	Reference	
Adobe	123	83 (67.5)	3.65 (2.34–5.75)	<0.0001
Palm products	20	19 (95.0)	33.44 (5.18–1394.30)	<0.0001
Type of floor inside house				
Concrete	280	44 (15.7)	Reference	
Sand/Clay	277	208 (75.1)	16.17 (10.40–25.24)	<0.0001
Toilet				
Pit latrine/WC	194	61 (31.4)	Reference	
None	363	191 (52.6)	2.42 (1.65–3.56)	<0.0001
Water Supply				
Exclusively well/tank	185	34 (18.4)	Reference	
Well and river	268	133 (49.6)	4.38 (2.76–7.01)	<0.0001
Exclusively river	104	85 (81.7)	19.86 (10.25–38.99)	<0.0001
Waste disposal				
Burnt	98	29 (29.6)	Reference	
Disposed on compound	326	138 (42.3)	1.75 (1.05–2.95)	0.025
Disposed outside compound	133	85 (63.9)	4.21 (2.32–7.68)	<0.0001
Electricity				
Yes	373	138 (37.0)	Reference	
No	184	114 (61.9)	2.77 (1.90–4.06)	<0.0001
**Animals on compound**				
Pigs				
Yes	111	101 (91.0)	19.73 (9.87–43.43)	<0.0001
No	446	151 (33.9)	Reference	
Dogs				
Yes	106	61 (57.6)	1.85 (1.18–2.90)	0.007
No	451	191 (42.4)	Reference	
Cats				
Yes	57	34 (59.7)	1.91 (1.05–3.50)	0.025
No	500	218 (43.6)	Reference	
Goats and/or chickens				
Yes	273	117 (42.9)	0.83 (0.58–1.17)	0.27
No	284	135 (47.5)	Reference	
**Knowledge. attitudes and practices**				
Knowledge on possible causes of tungiasis				
Sand/animals	416	141 (33.9)	Reference	
Other/does not know	141	111 (78.7)	7.22 (4.51–11.73)	<0.0001
Use of closed footwear				
Regularly	318	87 (27.3)	Reference	
No/Occasionally	239	165 (69.0)	5.92 (4.03–8.71)	<0.0001
Resting place				
Inside the house	299	60 (20.1)	Reference	
Outside the house	258	192 (74.4)	11.58 (7.63–17.06)	<0.0001
Use of soap for bathing				
Yes	496	203 (40.9)	Reference	
No	61	49 (80.3)	5.89 (2.99–12.45)	<0.0001
Type of prevention used				
Commercialized insecticides	53	5 (9.4)	Reference	
Water/other/none	504	247 (49.0)	9.27 (3.59–30.10)	<0.0001
Method of treatment used				
Treatment by paramedical personnel	85	14 (16.5)	Reference	
Self-treatment*	326	203 (62.3)	8.36 (4.42–16.71)	<0.0001
None	146	35 (24.0)	1.6 (0.77–3.45)	0.24

*: manipulation of lesions with non-sterile perforation instruments (such as needles. thorns). as well as application of hot oil. herbs etc.

In the multivariate logistic regression analysis, pigs on the compounds (adjusted OR = 18.0) and sandy floors inside houses (adjusted OR = 9.3) were the most important independent risk factors for infestation (Table 2). After controlling for confounding, the type of house was not an independent risk factor for infestation. Other modifiable factors independently associated with tungiasis included the resting place commonly used and the presence of cats. Individuals living in families that use regularly insecticides indoors had a lower chance to be infested. The regular use of closed footwear was also an independent protective factor (Table 2).

**Table 2:** Multivariate logistic regression analysis of factors associated with tungiasis.

	Adjusted OR (95% confidence interval)	*P* value
Presence of pigs on compound	17.98 (5.55–58.23)	<0.0001
Floor of sand or clay inside the house	9.33 (5.06–17.19)	<0.0001
Resting place outside house	7.14 (4.0–14.29)	<0.0001
Being ≤14 years or ≥40 years	5.02 (1.84–13.70)	0.002
Being illiterate/primary school not completed	4.16 (1.18–14.64)	0.026
Presence of cats on compound	4.16 (1.73–10.02)	0.001
Use of insecticides indoors	0.20 (0.05–0.83)	0.027
Use of soap for bathing	0.25 (0.08–0.77)	0.016
Use of water from well or tank	0.31 (0.16–0.59)	<0.0001
Regular use of footwear	0.34 (0.18–0.62)	<0.0001

The population attributable fractions for modifiable variables associated with tungiasis were: sand or clay floor inside the house (73.7%), resting commonly outside the house (65.5%), no regular use of closed footwear (51.1%), and pigs on the compound (37.9%; Table 3).

**Table 3:** Population attributable fractions of modifiable factors associated with tungiasis.

	Attributable risk	% exposed among cases	PAF
Floor of sand or clay inside the house	0,89	82.5	73.7
Common resting place outside house	0,86	76.2	65.5
Regular use of footwear	0,66	77.4	51.1
Presence of pigs on compound	0,94	40.1	37.9

## Discussion

Our data show that in Erekiti, a typical community in Western Nigeria with endemic tungiasis, several factors were independently associated with infestation by *Tunga penetrans*. In particular, sandy floor inside the house, behaviour (such as the common resting place and the use of closed footwear), as well as the presence of pigs on the compound contributed to an important extent to a high prevalence of tungiasis in the community. We identified the use of insecticides indoors and the use of soap, as well as the type of water supply as protective factors. In addition, the younger and older age groups were described as being most vulnerable for infestation.

So far, there is only one other study focussing on risk factors for tungiasis; this study was done in a poor fishing community in Brazil [16]. The importance of housing for the transmission dynamics has also been described in the Brazilian study. There, living in a house built on dunes, living in a house made of palm products, and having a floor of sand or clay inside the house were important risk factors for infestation in the multivariate analysis; adjusted odds ratios for these variables ranged from 1.9 to 4.7 [16]. Interestingly, in Nigeria the type of house was not an independent factor predisposing for infestation, but confounded by the type of floor inside the house and other factors. Thus, after controlling for confounding, the type of house *per se* did not predict infestation in Erekiti.

The findings of the present study corroborate our hypothesis made several years ago that the flea prefers sandy soil and shade for breeding and that, as an intervention, floors of houses could be cemented and streets paved [19]. This hypothesis is also confirmed by our finding that the resting place, which is commonly underneath a shady tree, is an important factor associated with infestation. We speculate that these places are preferred breeding sites of the flea, as there is abundant organic material for the larvae to feed on.

It is known that the animal reservoir plays an important role for transmission dynamics in endemic communities [20]. In particular, dogs, cats and rats have been described to be commonly infested [20]–[22], and several authors reported severe disease in pigs from different African countries, such as from São Tomé e Príncipe, Zaire, Cameroon and Tanzania [12], [23]–[26]. These studies emphasized the importance of pigs as animal reservoir of *T. penetrans*. Our data suggest that the presence of pigs on a family compound is an important predictor for human tungiasis and that pigs may be the most important animal reservoir this Nigerian community. Although we did not perform a formal prevalence study on domestic animals, we observed severely infested pigs in the community (Ugbomoiko, unpublished observation). Pigs did not play a role for transmission in Brazil, as in the studied community free-roaming pigs were absent [16]. Interestingly, in Brazil a significant reduction of attack rates in humans has been observed after the prohibition to let pigs roam freely in the community [19]. Similar intervention measures in Erekiti would probably reduce the prevalence of tungiasis in the community significantly. In contrast to the Brazilian study, we did not identify dogs, but cats to increase the prevalence in the community. In Brazil, dogs are commonly infested with prevalences reaching 67.0% in an urban slum [27].

The higher prevalence in children and the elderly in Erekiti is probably due to higher exposure and different disease-related behavior. Children play around (mostly barefooted) in the community, and the elderly have more difficulties to take out embedded fleas than young people. We observed in the community that skilful older children carry out flea extraction for their friends and younger children at school and that such assistance is rarely rendered to less skilful, poor sighted elder people [13].

We did not find any significant gender differences to predispose for infestation. Gender differences seem to differ from community to community. Whereas in some study areas, the male sex seems to be more vulnerable for tungiasis, in other areas, the females are more prone to infestation, or no gender differences have been observed [21], [28]–[32]. Thus, we speculate that gender differences are, similar to age, related to different exposure and disease-related behavior.

The use of proper footwear may decrease the prevalence in a community. According to our data, a consistent use of footwear would reduce infestation rates by about the half. However, economical, behavioural and cultural constraints may prohibit the intensive use of closed footwear in endemic communities in Western Africa.

Other socio-economic and behavioural factors found in this study, such as illiteracy, the type of water supply, and the use of soap, may be explained by an indirect relationship with tungiasis. For example, families with better access to water and using soap are prone to better hygiene standards. In addition, tungiasis can be regarded as a poverty-associated disease [1],[19], and improving sanitation and waste collection have been discussed as factors to reduce the incidence of tungiasis [19]. However, the effectiveness of these measures is difficult to predict, and they are more costly than cementing floors of houses, confining pigs to pigpens, and realizing health education.

Similar to our results, the use of insecticides inside houses has been described as a protective factor in the Brazilian study. This confirms further the notion of transmission indoors and also the need for *in vitro* studies on the effect of insecticides on pre-adult stages of *T. penetrans*.

The adjusted odds ratios and the population attributable fractions found in our study were very high for some variables. Although we have done an observational study, the strength of association, together with the biological plausibility of the discussed variables, increases the likelihood that the identified factors in fact have a causal relationship with tungiasis, even in the presence of unknown confounders. The identification of a limited number of obviously important factors helps to focus intervention measures on only a few variables which can be modified easily and without elevated costs. For example, cementing the floors of those houses with sandy or clay floor in the community would reduce prevalence of tungiasis by almost 75%. In addition, this measure will reduce transmission on the long run without any additional costs for the next years. Similar, confining pigs to pigpens and explaining to community members the location of breeding sites and areas of high transmission would reduce considerably the prevalence in the community. As a spin-off, the discussed measures probably reduce also the transmission of other parasitic diseases, such as neurocysticercosis, ancylostomiasis and strongyloidiasis.

In conclusion, the presence of tungiasis in the community is associated to an important extent with a set of a few modifiable variables. Effective and sustainable intervention measures addressing these factors need to be implemented in the study area, and in other communities throughout West Africa, to reduce the burden of this neglected tropical disease. An integrated approach combining the control of animal reservoirs, housing and environmental factors, and health education is necessary. Intervention measures need to be designed by an interdisciplinary team together with the affected communities.
